# Abernethy Malformation Type 2 and Biliary Atresia Coexistence: A Rare Cause of Infantile Liver Transplant

**DOI:** 10.5005/jp-journals-10018-1283

**Published:** 2019-02-01

**Authors:** Ezgi Yangın-Ergon, Nebahat Ermis, Ruya Colak, Burak Polat, Senem Alkan-Ozdemir, Meral Yıldız, Ferit Kulalı, Cigden Omur-Ecevit, Orkan Ergun, Sebnem Calkavur

**Affiliations:** 1 Department of Neonatology, Dr. BehCet Uz Pediatric Diseases and Surgery Education Research Hospital, Izmir, Turkey; 2 Department of Pediatric Radiology, Dr. BehCet Uz Pediatric Diseases and Surgery Education Research Hospital, Izmir, Turkey; 3 Department of Pediatric Gastroenterology, Dr. BehCet Uz Pediatric Diseases and Surgery Education Research Hospital, Izmir, Turkey; 4 Department of Pediatric Surgery and Transplantation, Ege University Hospital, Izmir, Turkey

**Keywords:** Abernethy malformation, Biliary atresia, Cholestasis, Liver transplant, Portal system.

## Abstract

**Introduction:**

Abernethy malformation is a very rare condition in which mesenteric venous blood is drained into systemic circulation by an extra hepatic portosystemic shunt. Here we present a case of a female in infantile period who is a liver transplant candidate with biliary atresia and ventricular septal defect (VSD) accompanying Abernethy malformation type 2.

**Case report:**

The patient who underwent Kasai operation at postnatal day 35 was identified as a liver transplant candidate in the postnatal month 6 due to growth retardation in the infantile period, insufficient weight gain and liver failure (portal hypertension and massive gastrointestinal bleeding).

**Conclusion:**

Abernethy malformation is divided into two groups as type 1 and type 2; type 2 shunts, which are less common, but more common in males, are not accompanied by other congenital anomalies. Due to the female gender, having biliary atresia and VSD, the development of liver failure at an early stage despite undergoing Kasai operation at the appropriate time, our case differs from the literature. In conclusion, this case is presented in order to indicate the proper and effective use of imaging methods in neonatal cholestasis cases.

**How to cite this article:** Ergon EY, Ermis N, Colak R, Polat B, Ozdemir S, Yildiz M, Kulali F, Ecevit C, Ergun O, Calkavur S. Abernethy Malformation Type 2 and Biliary Atresia Coexistence: A Rare Cause of Infantile Liver Transplant. Euroasian J Hepatogastroenterol, 2018;8(2):163-166.

## BACKGROUND

The veins forming the portal system are infused via the portal vein formed by the splenic vein and the superior mesenteric vein. Portal venous development is completed in the 4-10 weeks of embryonic life.^1^

John Abernethy first described portal venous congenital absence and superior mesenteric-vena cava inferior (VCI) shunt in 1793 as a postmortem in a 10-month-old female, and it was called Abernethy malformation.^2^ There are two types of shunts; type 1 is flowing of the entire portal blood to the VCI in relation to the congenital absence of portal venous, whereas type 2 is opening of the portal vein, which is intact but deflected, to the VCI by side-to-side or extrahepatic portosystemic shunt.^3^ Type 1 Abernethy malformation appears more frequently in females and is associated with other congenital anomalies such as cardiac defects (60%), biliary atresia (20%), polysplenia (20%), situs inversus (10%) and malrotation.^3^ Type 2 shunts are rarely encountered, mostly seen in males and generally unrelated to other congenital anoma-lies.^3^ The clinical findings of Abernethy malformation are various; while it can be seen in complicated cases such as hyperammonemia, hypoglycemia, hepatic encepha-lopathy, hepatopulmonary, and hepatorenal syndrome, it is occasionally seen in the asymptomatic course and incidentally diagnosed by imaging methods.^4,5^

Here, we present a case of a female with biliary atresia and VSD associated with Abernethy malformation type 2, who was diagnosed during the neonatal period and who was a candidate for liver transplant in the infantile period, because of differing from the literature and being a rare case.

## CASE DESCRIPTION

**Fig. 1:**
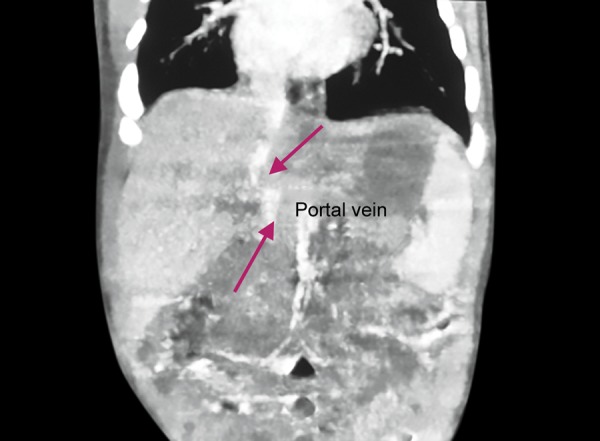
Radiosopic features of the portal vein which is intact but deflected

**Fig. 2:**
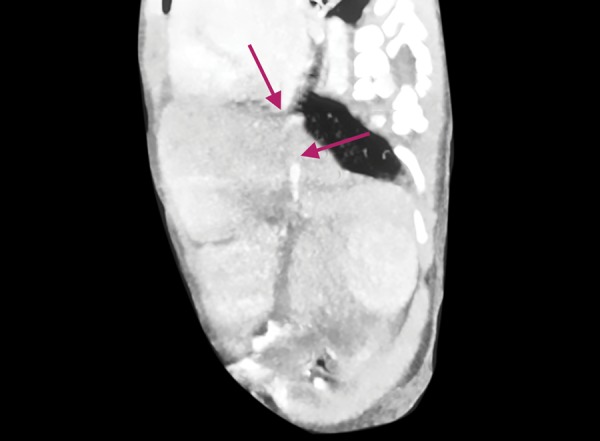
Abdominal CT angiography image of vena cava inferior proximal

As the first infant living in the third pregnancy of a 23-year-old mother, born by cesarean section in the weight of 2280 gm and having asymmetric intrauterine growth retardation, the female baby was referred to our unit with the diagnosis of neonatal cholestasis on the postnatal day 10, due to having acholic stool since birth and development of hyperbilirubinemia on the postnatal day two. The body weight was 2275 gr (3-10%), the height was 48 cm (10%), the head circumference was 33 cm (10-50%), the vital findings were; heart rate was: 124/min, blood pressure was: 70/48 (58) mm Hg, fever was: 36.7° C (axillary), and respiratory rate was: 52/min, spO2: 98%. In the physical examination, sclera and skin of the case were icteric and dirty-yellow looking; the case had hypertelorism, slanted eyes, and discrete hand fingers; and cardiac 3°/6 systolic murmur was detected. During the abdominal examination of the case who had no respiratory complaints, the liver and the spleen were not palpable. Laboratory investigations showed that aspartate aminotransferase (AST): 35 IU/L, alanine aminotransferase (ALT): 10 IU/L, albumin 3,3 gr/dL, alkaline phosphates (ALP) :280 IU/L, lactate dehydrogenase: 854 IU/L, blood glucose : 80 mg/dL ammonia: 80 mcg/dL values were normal; total bilirubin(t bil): 8mg/dL, direct bilirubin(d bil): 4.05 mg/dL, gamma-glutamyltransferase (GGT): 1185 IU/L values were high. Complete blood count, bleeding profile, blood gas, urinary examination were normal; infection criteria were negative. Abdominal ultrasonography (US) revealed a cystic structure without a real wall structure with an irregular contour of about 3 cm on the gladder ball wall. The lesion was evaluated in accordance with the pseudo biliary, and there was no evidence of choledochus in the case who had a finding of triangular cord at the level of the right-left hepatic venous junction. These findings were evaluated as compatible with biliary atresia. However, besides biliary atresia, the portal venous whose diameter was getting thinner was seen to open to the VCI without forming an intrahepatic branch after a short follow-up of portal hilus. MR angiography confirmed the diagnosis of biliary atresia, and abdominal CT angiography confirmed Abernethy malformation type 2 (Figs 1 and 2). The case, whose acolic stools were continuing, was followed by total oral feeding, although hypoglycemia was not observed, by pausing breastfeeding, and lactose-free formulas were used until galactosemia was excluded. A, D, E, and K vitamin supplements were increased due to cholestasis, and ursodeoxycholic acid treatment was started. In the echocardiography (ECO) of the case with cardiac murmur, whose peripheral pulses are open, and whose pre-postductal saturations and 4 extremity blood pressures showed no difference, inlet VSD was measured as 4 mm, secundumatriyoseptal defect (ASD) was 3 mm, and the stenosis gradient was measured as 40 mm Hg; and pulmonary stenosis and poststenotic dilatation were detected at valvular level. Spironolactone treatment for heart failure was initiated in the case. In the diagnosis of neonatal cholestasis separator, sepsis, viral infections, and hereditary metabolic diseases were excluded. Pheno-typic appearance was also evaluated in terms of Allagille Syndrome due to laboratory findings and ECO findings. Due to normal intrahepatic bile ducts in the abdominal US and no visible colloid, no posterior embryotoxic in the eye, no butterfly vertebrae in the vertebrae graphics, and because pulmonary stenosis was valvular but not peripheral in the ECO, the possibility of this diagnosis was excluded.

**Fig. 3:**
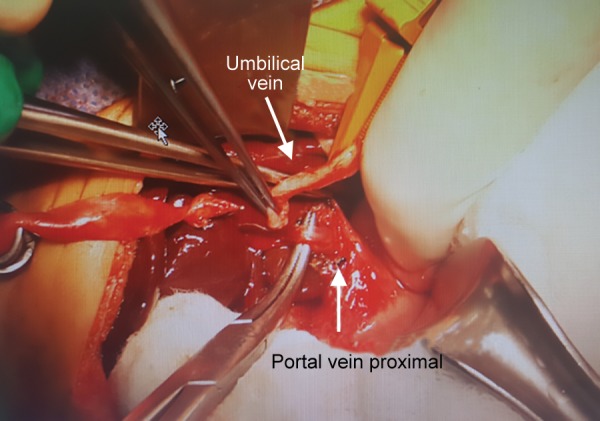
Surgery image of umbilical vein, portal vein was getting thinner at the hepatic hilus level

**Fig. 4:**
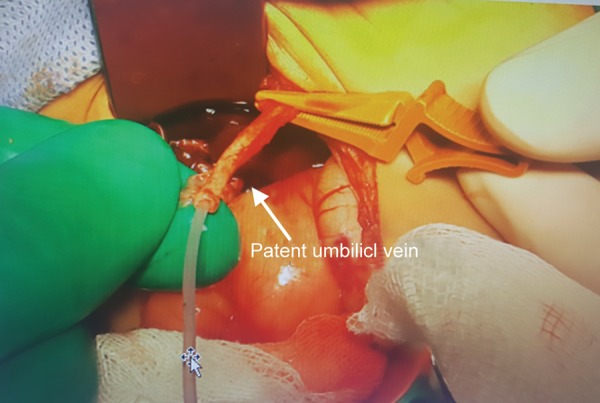
Patent umbilical vein

**Fig. 5:**
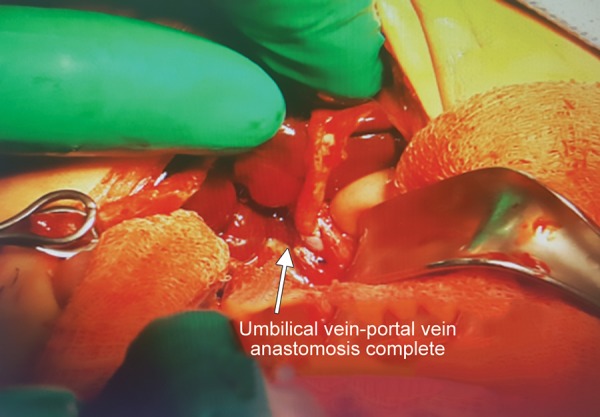
Umbilical vein-port

Exploration was performed to the case on the postnatal day 35. During the intraoperative follow-up, the gallbladder was atrophic, the liver did not have a cirrhotic appearance, but the main hepatic ducts were fibrotic, and portal vein was getting thinner at the hepatic hilus level and continued as an obliteration structure and also showed a slight extension to the VCI. Tip-to-tip anastomosis was performed between the portal vein and the umbilical vein by planning recanalization of the portal vein into the rex area of the left portal vein into which the umbilical vein flowed so that the portal vein could provide adequate blood supply. Intraoperative Doppler US showed that there was a slight flow in the umbilical vein; however, it was determined that sufficient revascularization could not be achieved because the segmented portal branches extending from the rex region into the liver were flebo-obliterated. The operation was completed with hepatic portoenterostomy (Kasai procedure) (Figs 3-5).

The two-month-old case who was re-hospitalized due to weight loss; had a body weight of 2950 gr (<3%) and a height of 50 cm, the infection criteria were negative, AST/ ALT: 325/144 IU/L, GGT: 102 IU/L, T bil/D bil: 1,44/ 8.9 mg/dL, bleeding profile was usual, nutrition was re-regulated by increasing calorie support.

The five-month-old case which applied to the emergency service due to bloody stool, bloody vomiting, the tendency to sleep had tachycardia, tachypnea, and abdominal distension. A patient with massive upper-lower GIS bleeding due to portal hypertension and hemorrhagic shock had hemoglobin: 5 gr/dL, INR:> 2,5, AST/ALT: 1202/544 IU/L, ALP: 677 IU/L, GGT: 43 IU/L, TIV/DIV: 15/7 mg/dL. Treatment for hypovolemic shock was performed and hemorrhage control was provided at the end of the second day.

Correspondingly, preparations were started for the case of a liver transplant candidate at postnatal month six due to the progression of chronic venous occlusion related to accompanying Abernethy malformation type 2 despite Kasai operation, and because the liver became fibrotic at the infantile stage and liver failure developed.

## DISCUSSION

Congenital absence of portal vein; is a rare condition in which intestinal and splenic venous blood bypasses the liver and drains directly into the systemic circulation with a venous shunt.^6^ In the intrauterine embryonic period, connections between the right and left vitelline venules occur at the end of the 4th week, and intrahepatic and extrahepatic portal veins develop from these connections. As a result of the inadequacy of anastomosis, portosystemic and portoportal shunts occur. There are two types of portosystemic shunt anomalies, and there is no intrahepatic portal vein in type 1 shunt, and there is a complete end-to-side shunt. Type 1 shunts are divided into two subgroups as type 1A; superior mesenteric vein and splenic vein flows into the VCI, the iliac vein, and the renal veins separately, and as type 1B; superior mesenteric vein and splenic vein are joined together to form a short extra hepatic portal vein.^7^ In type 2 shunts, there is a patented intrahepatic portal vein and there is a partial side-to-side shunt in the portal vein.^6^ The literature does not usually make this distinction, and Abernethy malformation is used synonymously with congenital absence of portal vein.^6^

In our case, after a short follow-up, portal vein is opened to VCI through the portal hilus without forming an intrahepatic branch, and the diameter gets thinner; the shunt has been evaluated as Abernethy malformation type 2. Identification of the malformation type is important in terms of determining the treatment algorithm. Concomitant hepatopulmonary syndrome, liver dysfunction, type 1 malformations due to focal nodular hyperplasia may require tumor resection and/ or liver transplantation.^8^ Type 2 malformations in which portosystemic encephalopathy or portal hypertension is observed can be closed by surgical ligation or invasive embolization.^8^ Asymptomatic cases diagnosed incidentally by imaging methods can be followed up with intermittent controls.^8^

Type 1 shunts are associated with other congenital anomalies such as cardiac defects, biliary atresia, poly-splenia, and liver tumors, defined only in females, while type 2 shunts are; rarely seen in males and independent of other congenital anomalies.^3^ Even though it is a type 2 shunt, our case is different from the literature because of the presence of biliary atresia, VSD and valvular pulmonary stenosis, and the female gender.

Abernethy malformation alone does not create an indication for liver transplantation. If there are no concomitant coexisting anomalies, it can be detected incidentally in many cases during surgery or during imaging methods such as US, CT or MR for liver disease.^9,10^ With regard to the literature, live donor liver transplantation due to Abernethy malformation has been performed in 11 cases in the pediatric population so far.^11-13^ Kasai operation has been performed during the neonatal period in cases accompanied by biliary atresia, and liver transplantation has been performed to these cases, even in the case of biliary atresia, in the late childhood or adolescent age.^11^ In our case, although Kasai procedure was performed in the neonatal period and portal vein reconstruction was performed at the same session, due to the accompanying Abernethy malformation Type 2, portal vein hypertension and liver fibrosis developed when the case was six months old and became a transplant candidate in the infantile period.

Despite the expectation of an increase in postoperative morbidity and mortality in association with biliary atresia, Abernethy malformation, and cardiac defects, this association does not cause a contraindication for liver transplantation. In earlier periods of life, transplant requirement may develop. Patients with neonatal cholestasis should be assessed using advanced imaging methods during the presence of biliary atresia, one of the first anatomic disorders that should be excluded, in terms of the additional vascular anomalies that accompany this condition, because it changes the course of the disease, and the success of Kasai operation, and it should be kept in mind that there may be portosystemic and portoportal shunts affecting the liver blood flow.

## CONCLUSION

As a result in neonatal cholestasis cases, the rapid and accurate diagnosis should be made with advanced imaging methods, and anatomical portal vascularity should be evaluated in detail. In the case of identification of Abernethy malformation accompanied by biliary atresia, it is essential to monitor the cases in well-equipped centers where living donor liver transplantation can be performed, considering postoperative complications.
